# Oxygen saturation imaging elucidates tumor heterogeneity in gastric cancer

**DOI:** 10.1002/deo2.70077

**Published:** 2025-02-21

**Authors:** Tatsunori Minamide, Nobuhisa Minakata, Riu Yamashita, Shingo Sakashita, Yusuke Yoda, Akihiro Ohashi, Masato Aoshima, Susumu Kobayashi, Tomonori Yano

**Affiliations:** ^1^ Department of Gastroenterology and Endoscopy National Cancer Center Hospital East Chiba Japan; ^2^ Department of Gastroenterology IMSUT Hospital, The Institute of Medical Science, The University of Tokyo Tokyo Japan; ^3^ Division of Translational Informatics, Exploratory Oncology Research & Clinical Trial Center National Cancer Center Chiba Japan; ^4^ Department of Pathology and Clinical Laboratories National Cancer Center Hospital East Chiba Japan; ^5^ Division of Translational Genomics, Exploratory Oncology Research & Clinical Trial Center National Cancer Center Chiba Japan; ^6^ Department of Medicine, Beth Israel Deaconess Medical Center Harvard Medical School Boston USA

**Keywords:** endoscopy, heterogeneity, oxygen saturation, stomach neoplasms, tumor hypoxia

## Abstract

Oxygen saturation imaging is a new technology that determines biological features from the perspective of oxygen concentration. Therefore, this exploratory study aimed to evaluate the biological implications of oxygen saturation imaging and further assess tumor heterogeneity in gastric cancer. Biopsy samples were selectively obtained from treatment‐naïve patients with gastric cancer under real‐time oxygen saturation imaging. Tissue oxygen saturation level calculations, immunohistochemistry, and RNA sequencing were performed. The mean tissue oxygen saturation levels at the sampling sites were 32.2%, 70.8%, and 56.2% for hypoxic, hyperoxic, and non‐tumor areas, respectively, with significant differences between each pair. CD‐31 and glucose transporter 1 protein expression were significantly upregulated in hypoxic tumors. Comprehensive transcriptomic analysis revealed enriched biological processes related to the regulation of insulin‐like growth factor transport and uptake by insulin‐like growth factor‐binding proteins in hypoxic tumors and the type I interferon signaling pathway in hyperoxic tumors. Oxygen saturation imaging has the potential to clarify hypoxia‐induced heterogeneity in gastric cancer from both clinical and fundamental perspectives.

## INTRODUCTION

Hypoxia is a common solid tumor characteristic caused by uncontrolled cell proliferation and incomplete angiogenesis.^1^ By upregulating the master transcription factor hypoxia‐inducible factor‐1 (HIF‐1), the hypoxic microenvironment plays a pivotal role in tumor progression by promoting biological processes, including angiogenesis, glucose metabolism, invasion, and metastasis.^2^, ^3^ Intratumoral hypoxia is associated with oncological therapeutic resistance of various solid tumors; however, the biological background of oxygen heterogeneity has not been elucidated, particularly in gastric cancer, which harbors high biological heterogeneity.^4^

Oxygen saturation (OS) imaging, a novel endoscopic gastrointestinal technique, allows real‐time visualization of saturation of tissue oxygen (StO_2_) levels by displaying them as pseudocolor images on the mucosal surface. The OS imaging system is based on the spectral differences between oxyhemoglobin and deoxyhemoglobin. As previously described, we used two laser diodes with different wavelengths (445 and 473 nm).^5^ While various endoscopic approaches have been developed for the accurate detection and qualitative diagnosis of gastrointestinal cancer, OS imaging is a new technology that determines biological features from an oxygen concentration perspective. Therefore, this exploratory study aimed to evaluate the biological implications of OS imaging and assess intratumoral heterogeneity in gastric cancer based on OS imaging with selective tissue biopsies.

## PROCEDURE OR TECHNIQUE

### Study design and patients

This observational study was conducted between November 2019 and April 2020 at a tertiary referral center in Japan. The inclusion criterion was patients with treatment‐naïve gastric cancer scheduled to undergo esophagogastroduodenoscopy (EGD) for endoscopic lesion diagnosis and tumor sampling. The exclusion criterion was patients who could not undergo EGD with accurate StO_2_ measurement by OS imaging because of adherent substances, including blood and white coating.

Written informed consent for study participation was obtained from all patients before the endoscopic procedures. This study was approved by the Institutional Review Board of the National Cancer Center Hospital East (registration number 2019–076) and conducted according to the 1964 Declaration of Helsinki and its later amendments.

### Clinicopathological data collection

The following clinicopathological data were collected from the patient's electronic medical records: age, sex, tumor location, macroscopic tumor type, tumor size, histopathological type, and clinical stage.

### Endoscopic procedure

**FIGURE 1:**
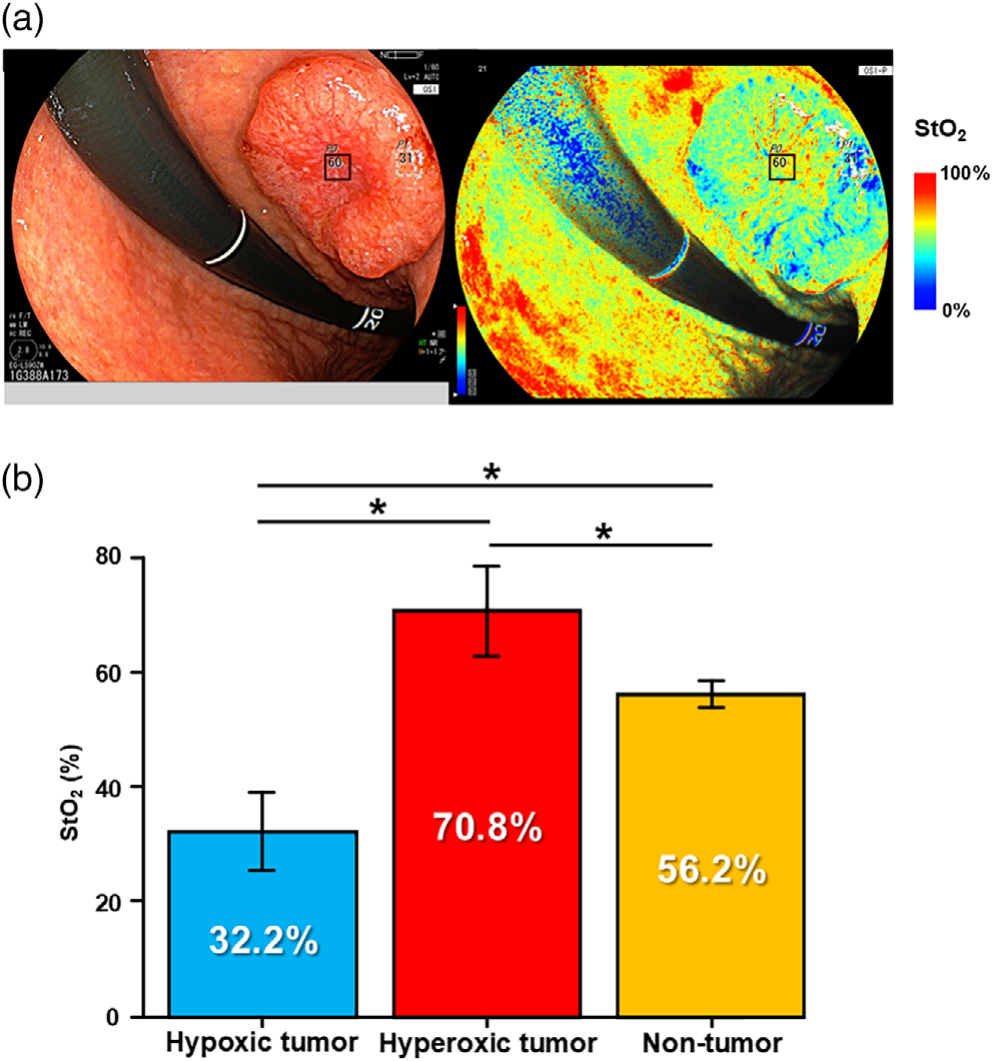
Oxygen saturation imaging and tissue oxygen saturation measurement. (a) Representative endoscopic images acquired using oxygen saturation imaging. In this case, tissue oxygen saturation (StO_2_) levels for hypoxic (white dashed box) and hyperoxic tumor areas (black box) were 31% and 60%, respectively. (b) StO_2_ levels in hypoxic tumors, hyperoxic tumors, and non‐tumor areas, from which biopsy samples were obtained using real‐time oxygen saturation imaging. *p < 0.05.

All EGDs were performed for OS imaging using the LASEREO system and EG‐L590ZW (FUJIFILM Co., Tokyo, Japan). Only board‐certified endoscopists performed the EGDs. Endoscopic images of white light and the OS were captured simultaneously and projected onto two screens. Real‐time OS imaging was projected as a pseudocolor endoscopic image depending on the StO_2_ levels, enabling the endoscopists to predict StO_2_ levels from the color differences (Figure 1a). Two biopsy samples were selectively obtained from each non‐tumor area and hypoxic and hyperoxic tumor area (one each for immunohistochemistry and RNA sequencing), as determined by OS imaging. Since there was no clear definition of hypoxic and hyperoxic tumors under OS imaging, the endoscopists determined relative hypoxic and hyperoxic areas within the same tumor at the time of observation. After thoroughly washing and removing mucus, bile, blood, and other adherent materials that could affect OS imaging,^6^ a biopsy was taken from a non‐tumor area at least 1 cm away from the tumor. After EGD completion, the obtained endoscopic images were used to measure StO_2_ levels at the sampling sites using dedicated software developed by FUJIFILM Co. StO_2_ levels were calculated as the average value within a small square at the sampling site.

### Immunohistochemistry

Immunohistochemistry was performed on sections (5 µm thickness) dissected from formalin‐fixed paraffin‐embedded biopsy samples. After deparaffinization, rehydration, and antigen retrieval using microwave heating, the endogenous peroxidase activity was blocked using 0.3 % hydrogen peroxide/methanol for 30 min at room temperature. The sections were incubated with primary antibodies against CD‐31 (M0823; Dako, Glostrup, Denmark) at a dilution of 1:200 and glucose transporter 1 (GLUT‐1; sc‐377228; Santa Cruz Biotechnology, CA, USA) at the same dilution overnight at 4°C. Subsequently, the sections were incubated with horseradish peroxidase‐conjugated secondary antibodies for 30 min at room temperature. Finally, the sections were stained with diaminobenzidine solution for 15 min, followed by counterstaining with hematoxylin. Three rectangular fields (0.1 mm^2^) were randomly selected under a light microscope and processed using a computer‐aided automatic image analysis system (HALO; Indica Labs, NM, USA). For CD‐31, the stain‐positive area per field of view was calculated. For GLUT1, the H‐score was used for semi‐quantitative expression assessment. The H‐score was calculated by grading the staining intensity into four levels (none, weak, moderate, and strong staining), multiplying the cell percentage in each grade by 0–3, and then summing the results.

### RNA sequencing analysis

Total RNA was extracted from the biopsy samples using the RNeasy Mini Kit (Qiagen, USA), according to the manufacturer's instructions. Complementary DNA library construction and sequencing were performed on the BGISEQ‐500 platform. High‐quality reads were aligned with the human reference genome (GRCh38) using spliced transcript alignment to a reference (STAR) software. The expression level of each gene was normalized to the transcripts per kilobase of the exon model per million mapped reads using RNA sequencing with expectation maximization. ComBat‐seq was used to eliminate batch effects. For transcriptomic analysis, differentially expressed genes were selected between hypoxic and hyperoxic tumors according to the combination of the absolute value of log2‐Ratio > 1 and false discovery rate < 0.01. Gene ontology analysis of differentially expressed genes was performed using Metascape (http://metascape.org).

### Statistical analysis

Categorical data were expressed as frequencies (%). Continuous variables were expressed as medians with ranges and compared using Student's t‐test or one‐way analysis of variance with appropriate Bonferroni's post‐hoc tests. All statistical analyses were performed using EZR (Saitama Medical Center, Jichi Medical University, Saitama, Japan), a graphical user interface for R (R Foundation for Statistical Computing, Vienna, Austria). Statistical significance was set at *P* < 0.05.

## RESULTS

We recruited six patients with treatment‐naïve gastric cancer who underwent EGD for lesion diagnosis using OS imaging. Most cases were of advanced and pathologically differentiated cancer types (Table 1). All lesions were reddish type 2 or 3 ulcerative lesions. To confirm whether pseudocolor endoscopic images by OS imaging correlated with StO_2_ levels, we calculated StO_2_ levels at the sampling sites, which endoscopists determined to be hypoxic or hyperoxic using real‐time OS imaging (Figure 1a). We observed a clear correlation between StO_2_ levels and sampling sites, with means of 32.2 %, 70.8 %, and 56.2 % for hypoxic, hyperoxic, and non‐tumor areas, respectively. This finding demonstrates significant differences between the pairs (Figure 1b). Real‐time OS imaging was confirmed to be beneficial in estimating StO_2_ levels, highlighting significant intratumoral oxygen heterogeneity.

**TABLE 1:** Baseline clinicopathological characteristics of six patients with gastric cancer.

Characteristic	*n* = 6
Age, median, years (range)	73 (60–84)
Sex, *n* (%)	
Male	4 (66.7)
Female	2 (33.3)
Location, *n* (%)	
Upper third	2 (33.3)
Middle third	3 (50.0)
Lower third	1 (16.7)
Macroscopic type, *n* (%)	
Type 2	4 (66.7)
Type 3	2 (33.3)
Size, median, mm (range)	48 (28–100)
Histopathological type, *n* (%)	
Differentiated	5 (83.3)
Undifferentiated	1 (16.7)
Stage, *n* (%)	
I	1 (16.7)
III	4 (66.7)
IV	1 (16.7)
*Helicobacter pylori* infection status, *n* (%)	
Uninfected	1 (16.7)
Infected	4 (66.7)
Unknown	1 (16.7)

**FIGURE 2:**
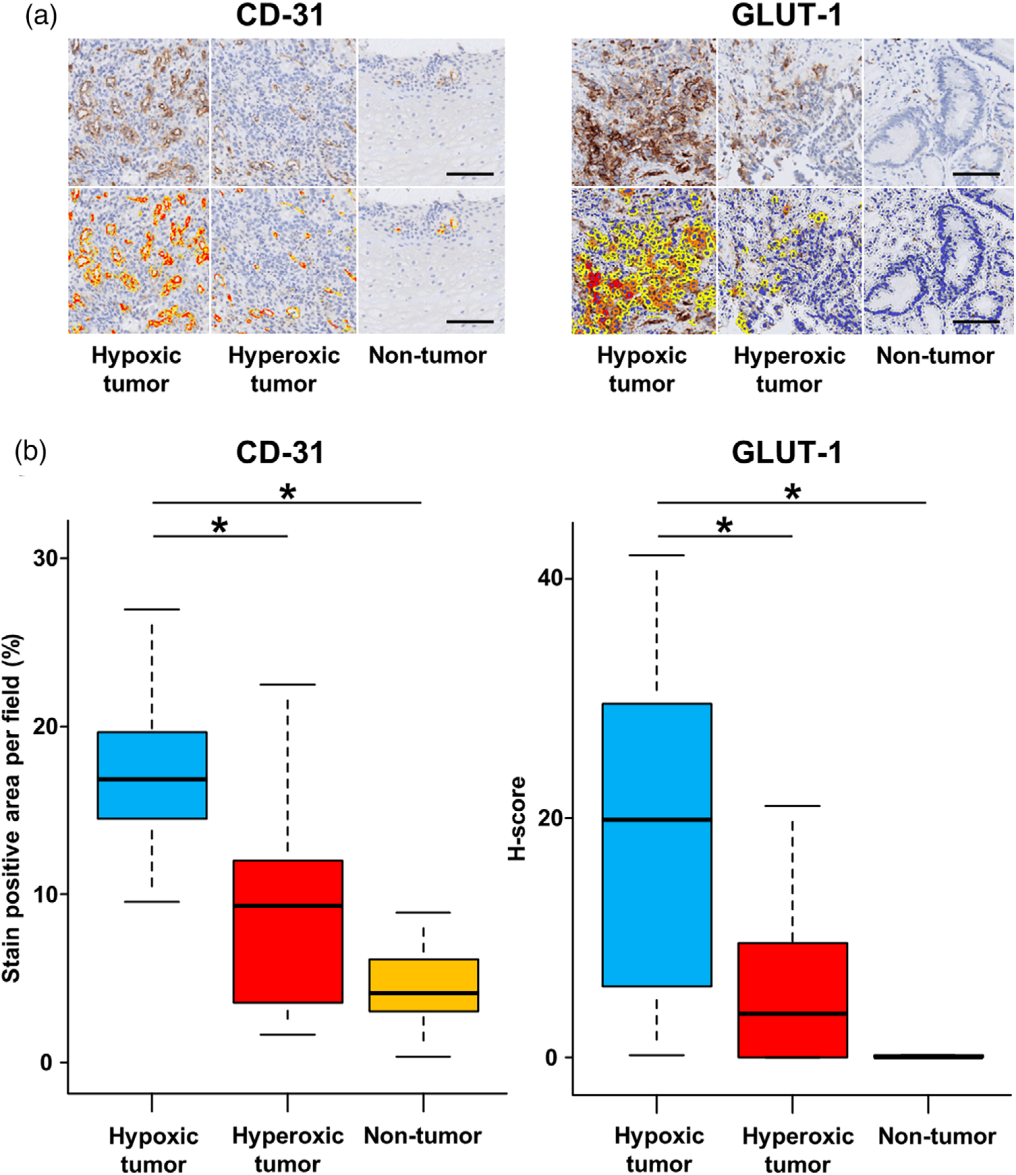
Immunohistochemistry based on oxygen saturation heterogeneity. (a) Representative immunohistochemical staining images of biopsy samples from gastric cancers with CD‐31 and glucose transporter 1 (GLUT‐1). The top row shows the stained images, and the bottom row shows the resulting images from the computer‐assisted analysis. The scale bars correspond to 100 µm. (b) Quantitative and semi‐quantitative immunohistochemistry image evaluation of CD‐31 and GLUT‐1 in hypoxic tumors, hyperoxic tumors, and non‐tumor areas. The stain‐positive areas per field of view for CD‐31 (left) and the H‐score for GLUT‐1 (right) were assessed. *p < 0.05.

To determine the biological significance of OS imaging, we investigated the protein expressions of CD‐31 and GLUT‐1 using immunohistochemistry (Figure 2a). CD‐31 is a microvascular endothelial cell marker. GLUT‐1 is responsible for glucose uptake, and the HIF‐1 cascade induces its transcription under hypoxic conditions.^3^ Notably, the hypoxic tumors had significantly greater CD‐31 stain‐positive areas per field of view than the hyperoxic tumors and non‐tumor areas (16.8 % vs. 9.4 % vs. 4.1 %; Figure 2b). Additionally, the H‐scores for GLUT‐1 were significantly higher in hypoxic tumors than in hyperoxic tumors and non‐tumor areas (19.9 vs. 3.7 vs. 0.0). These findings suggest a correlation between OS imaging and the upregulation of biological processes in hypoxic tumors.

**FIGURE 3:**
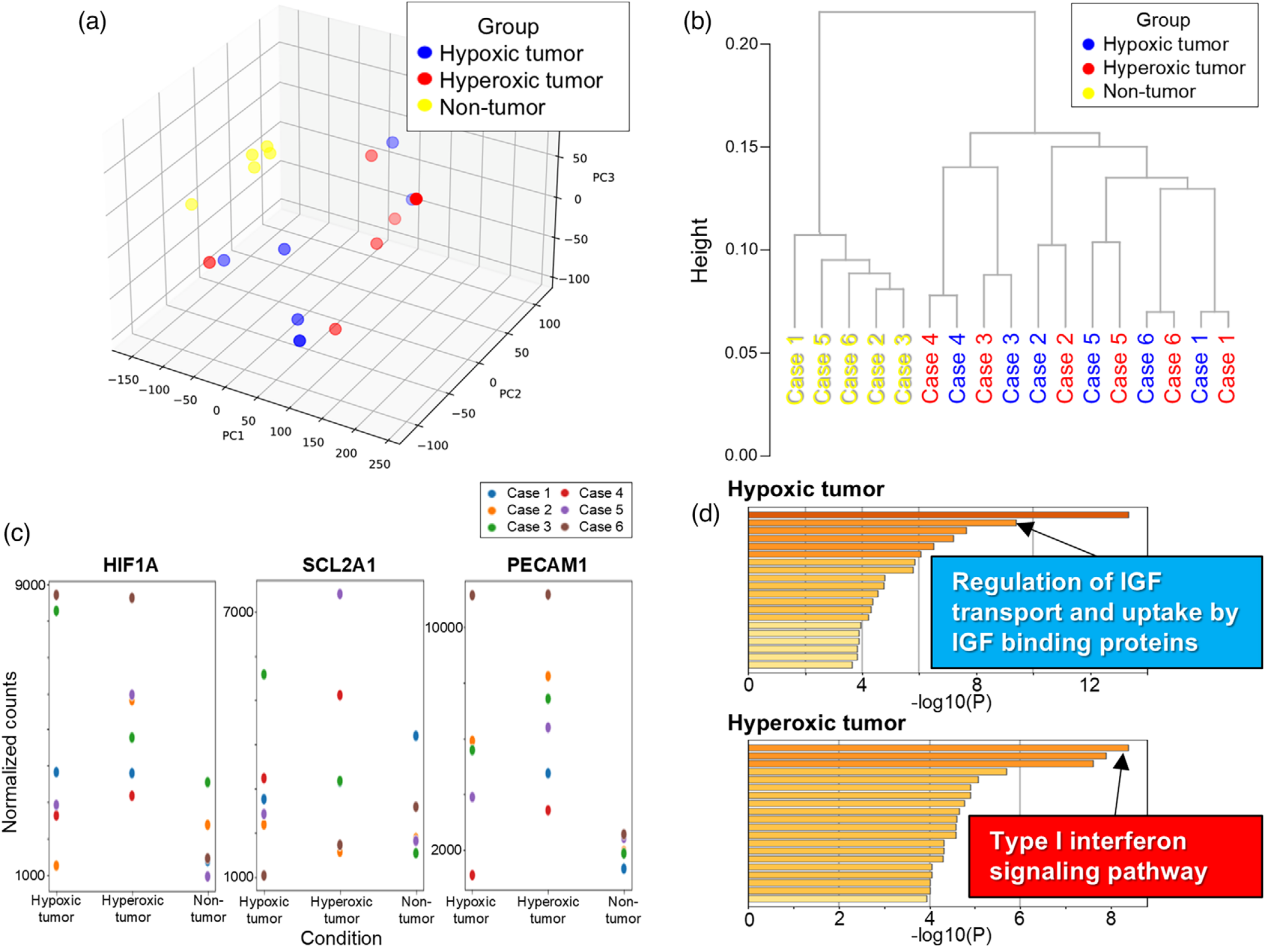
Comprehensive transcriptomic analysis depending on oxygen saturation heterogeneity. (a) Principal component analysis and (b) hierarchical clustering analysis of the comprehensive gene expression profiles for each area using oxygen saturation imaging. (c) Expression of HIF1A, SCL2A1, and PECAM1, representative genes of the HIF‐1 cascade. (d) Gene ontology analysis of differentially expressed genes in hypoxic (upper panel) and hyperoxic (lower panel) tumors. Only the biological processes of interest were annotated. The non‐tumor area of Case 4 was not analyzed because RNA of sufficient quality could not be obtained.

RNA sequencing was performed on each biopsy sample to compare comprehensive gene expression profiles using OS imaging. Quality control data are presented in Table S1. The principal component analysis demonstrated a significant transcriptomic difference between tumor and non‐tumor tissues, confirming exact tumor sampling (Figure 3a). In addition, the hierarchical clustering analysis showed greater interpatient heterogeneity than intrapatient heterogeneity (Figure 3b). Figure 3c shows the expression of HIF1A, SCL2A1, and PECAM1, representative genes of the HIF‐1 cascade. However, the correlation between groups was less pronounced compared to immunohistochemical staining. Gene ontology analysis of the differentially expressed genes (Table S2) revealed that distinct pathways were activated in response to StO_2_ (Figure 3d and Table 2). In hypoxic tumors, the biological processes related to the regulation of insulin‐like growth factor (IGF) transport and uptake by IGF‐binding proteins were enriched. In hyperoxic tumors, biological processes related to the type I interferon signaling pathway were enriched. Collectively, our comprehensive transcriptomic gastric cancer analysis delineated the biological differences between hypoxic and hyperoxic tumors based on selective tissue sampling using OS imaging.

**TABLE 2:** Results of gene ontology analysis of the differentially expressed genes.

Hypoxic tumor		
Term	Description	‐Log10 (*p*)
ko05322	Systemic lupus erythematosus	13.3467011172
R‐HSA‐381426	Regulation of insulin‐like growth factor (IGF) transport and uptake by insulin‐like growth factor binding proteins (IGFBPs)	9.3896787623
GO:0007268	Chemical synaptic transmission	7.6538316517
GO:0098742	Cell‐cell adhesion via plasma‐membrane adhesion molecules	7.1964942062
GO:2000609	Regulation of thyroid hormone generation	6.5073450224
R‐HSA‐977225	Amyloid fiber formation	6.0569753008
R‐HSA‐174577	Activation of C3 and C5	5.8466870726
hsanan01	Drug metabolism	5.7734696832
hsa05032	Morphine addiction	4.7855927151
M5885	NABA MATRISOME ASSOCIATED	4.7485822404
GO:0002576	Platelet degranulation	4.5394056396
GO:0042060	Wound healing	4.3681574021
GO:0019730	Antimicrobial humoral response	4.30879
GO:0043269	Regulation of ion transport	4.21892
GO:0015850	Organic hydroxy compound transport	3.937614757
GO:0015893	Drug transport	3.888465331
GO:0071248	Cellular response to metal ion	3.88206399
GO:0007389	Pattern specification process	3.809581543
GO:0016338	Calcium‐independent cell‐cell adhesion via plasma membrane cell‐adhesion molecules	3.807009539
GO:0045995	Regulation of embryonic development	3.626085612

Hyperoxic tumor		
Term	Description	‐log10 (*p*)
GO:0060337	Type I interferon signaling pathway	8.38375127
GO:0006638	neutral lipid metabolic process	7.879192364
GO:0050892	Intestinal absorption	7.599153023
GO:0006066	Alcohol metabolic process	5.684823922
GO:0016266	O‐glycan processing	5.069585304
GO:0048608	Reproductive structure development	4.901162304
GO:0048732	Gland development	4.901162304
GO:0009410	Response to xenobiotic stimulus	4.758520467
GO:0008610	Lipid biosynthetic process	4.657150014
GO:0060290	Transdifferentiation	4.584290413
M106	PID HNF3B PATHWAY	4.567164798
GO:0031018	Endocrine pancreas development	4.567164798
GO:0021987	cerebral cortex development	4.30303585
GO:0002065	Columnar/cuboidal epithelial cell differentiation	4.30303585
GO:0002685	Regulation of leukocyte migration	4.292608622
GO:0048469	Cell maturation	4.043451304
GO:1903532	Positive regulation of secretion by cell	4.042071918
GO:0010960	Magnesium ion homeostasis	4.000161862
GO:0035404	Histone‐serine phosphorylation	4.000161862
GO:0120162	Positive regulation of cold‐induced thermogenesis	3.921200439

## DISCUSSION

To our knowledge, this is the first study to evaluate OS imaging focusing on oncological implications, particularly intratumoral heterogeneity. We have previously reported the usefulness of this technology in assessing decreased blood flow, that is, acute hypoxia.^5^, ^7^ Our data demonstrated that real‐time OS imaging also serves as a powerful endoscopic tool for precise intratumoral hypoxia assessment, which has long been difficult to determine within the human body.

Compared to existing imaging techniques, including needle electrode systems and positron emission tomography,^8^ OS imaging is a minimally invasive and high‐resolution technique for assessing intratumoral hypoxia. Moreover, this technique does not require additional devices or dyes; it requires the endoscopist to push a button on the endoscope, allowing for rapid and comprehensive assessment of heterogeneous StO_2_ levels. Besides OS imaging, various other endoscopic developments for measuring StO_2_ levels have been attempted.^9^, ^10^, ^11^, ^12^ Innovative technologies were applied, such as thermal imaging, photoacoustic imaging, and hyperspectral imaging. Although some have been validated in vivo, there are few reports of their actual usefulness in the human body compared to OS imaging.

Under hypoxic conditions, tumor cells adjust their metabolic programs and promote angiogenesis and metastasis, as well as DNA repair and immune escape by activating HIF‐1 expression.^2^, ^3^, ^13^ HIF‐1 induces vascular endothelial growth factor and basic fibroblast growth factor, which increases vascular endothelial cells and enhances vascular permeability.^14^, ^15^ As hypoxia was reported to be associated with GLUT‐1 expression and ^18^F‐fluorodeoxyglucose uptake in disseminated peritoneal tumors,^16^ HIF‐1 transcription also results in upregulation of glucose transporters. However, hyperoxia has been reported to activate the antitumor immune cells by weakening the hypoxia–A2‐adenosinergic immunosuppression.^17^

Herein, we elucidated the biological implications of OS imaging using protein and gene expression analyses based on selective tissue biopsies from hypoxic and hyperoxic tumors. Intriguingly, protein expression analysis revealed that hypoxic areas, as determined by OS imaging, correlate with angiogenesis and glucose metabolism enhancements, which are associated with tumor hypoxia.^2^, ^3^, ^18^ Therefore, OS imaging in clinical settings reflects the biological dynamics of gastric cancer. Furthermore, the comprehensive transcriptomic analysis demonstrated that tumor heterogeneity depends on StO_2_ levels and the characteristic biological program enrichment related to intratumoral hypoxia and hyperoxia. The IGF pathway reportedly contributes to treatment resistance in lung cancer via hypoxia,^19^ and recent studies have revealed that the type I interferon pathway is downregulated in several hypoxia‐induced cancers. Our findings and these data may provide clues to elucidate hypoxia‐induced resistance mechanisms using OS imaging.^20^

Although the limitations of this exploratory study are that it included only a small number of cases and lacked a clear definition of hypoxic and hyperoxic areas, our observations will allow OS imaging to be a revolutionary tool in future studies focusing on tissue oxygen heterogeneity in gastrointestinal cancers from both clinical and basic medical perspectives. We believe that OS imaging has the potential to clarify the molecular mechanisms of the hypoxic tumor microenvironment, predict therapeutic effects, and resolve treatment resistance due to oxygen heterogeneity.

## CONFLICT OF INTEREST STATEMENT

Tomonori Yano is Deputy Editor‐in‐Chief of Digestive Endoscopy and received a research grant from FUJIFILM for this study, which provided the oxygen saturation imaging endoscopy system used in this research. The remaining authors have no conflict of interest.

## ETHICS STATEMENT

Approval of the research protocol by an Institutional Reviewer Board: The Institutional Review Board of the National Cancer Center Hospital East approved the research protocol (registration number 2019–076).

## PATIENT CONSENT STATEMENT

Written informed consent for study participation was obtained from all patients before the endoscopic procedures.

## CLINICAL TRIAL REGISTRATION

N/A.

## Supporting information




**TABLE S1** Quality control data of RNA sequencing.

**TABLE S2** Top 20 differentially expressed genes in hypoxic tumor compared to hyperoxic tumor.
