# Supplementary material for: Oxygen saturation imaging elucidates tumor heterogeneity in gastric cancer
Source: DEN Open. 2025 Feb 21;5(1):e70077. doi: 10.1002/deo2.70077 (PMC11843471; doi:10.1002/deo2.70077)
Supplement: Supplementary file 1 — TABLE S1 Quality control data of RNA sequencing. [file DEO2-5-e70077-s002.docx]

**Supplementary Table 1. Quality control data of RNA sequencing.**

| Sample | Total raw reads (M) | Total clean reads (M) | Total clean bases (Gb) | Clean reads Q20 (%) | Clean reads Q30 (%) | Clean reads ratio (%) |
| --- | --- | --- | --- | --- | --- | --- |
| Case 1_Hypo | 89.95 | 83.52 | 8.35 | 98.36 | 92.32 | 92.85 |
| Case 1_Hyper | 92.45 | 85.44 | 8.54 | 98.32 | 92.17 | 92.41 |
| Case 1_Non | 92.45 | 84.91 | 8.49 | 98.34 | 92.31 | 91.84 |
| Case 2_Hypo | 89.95 | 83.53 | 8.35 | 98.28 | 92.14 | 92.86 |
| Case 2_Hyper | 92.45 | 85.53 | 8.55 | 98.47 | 92.82 | 92.52 |
| Case 2_Non | 92.45 | 85.36 | 8.54 | 98.26 | 91.87 | 92.32 |
| Case 3_Hypo | 89.95 | 83.48 | 8.35 | 98.3 | 92.05 | 92.81 |
| Case 3_Hyper | 89.95 | 83.46 | 8.35 | 98.38 | 92.37 | 92.78 |
| Case 3_Non | 92.45 | 85.55 | 8.56 | 98.15 | 91.57 | 92.54 |
| Case 4_Hypo | 97.45 | 89.95 | 9.00 | 97.43 | 89.82 | 92.30 |
| Case 4_Hyper | 99.95 | 91.79 | 9.18 | 97.50 | 90.05 | 91.83 |
| Case 5_Hypo | 94.95 | 87.87 | 8.79 | 97.50 | 89.85 | 92.54 |
| Case 5_Hyper | 94.95 | 88.06 | 8.81 | 97.45 | 89.59 | 92.74 |
| Case 5_Non | 94.95 | 88.51 | 8.85 | 97.48 | 89.73 | 93.22 |
| Case 6_Hypo | 99.95 | 91.79 | 9.18 | 97.36 | 89.53 | 91.84 |
| Case 6_Hyper | 94.95 | 87.85 | 8.79 | 97.48 | 89.88 | 92.52 |
| Case 6_Non | 99.95 | 91.26 | 9.13 | 97.30 | 89.28 | 91.31 |

Hypo, hypoxic tumor; Hyper, hyperoxic tumor; Non, non-tumor.
