# Supplementary material for: Oxygen saturation imaging elucidates tumor heterogeneity in gastric cancer
Source: DEN Open. 2025 Feb 21;5(1):e70077. doi: 10.1002/deo2.70077 (PMC11843471; doi:10.1002/deo2.70077)
Supplement: Supplementary file 2 — TABLE S2 Top 20 differentially expressed genes in hypoxic tumor compared to hyperoxic tumor. [file DEO2-5-e70077-s001.docx]

**Supplementary Table 2. Top 20 differentially expressed genes in hypoxic tumor compared to hyperoxic tumor.**

| SYMBOL | logFC | logCPM | LR | PValue | FDR |
| --- | --- | --- | --- | --- | --- |
| *WDR72* | 12.92177 | 4.006459 | 143.4746 | 4.63E-33 | 1.11E-28 |
| *XIST* | -8.70745 | 5.676972 | 112.6239 | 2.61E-26 | 2.08E-22 |
| *NOTUM* | 9.191147 | 4.786436 | 91.75408 | 9.81E-22 | 4.70E-18 |
| *GNG4* | 6.765353 | 3.823072 | 91.23022 | 1.28E-21 | 4.82E-18 |
| *NKX6-3* | -12.6067 | 2.555957 | 91.0394 | 1.41E-21 | 4.82E-18 |
| *GATA4* | -6.29622 | 6.436564 | 77.98822 | 1.04E-18 | 3.10E-15 |
| *PAX6* | -11.9088 | 1.860731 | 76.81397 | 1.88E-18 | 5.00E-15 |
| *KRT8P36* | -12.2849 | 2.357463 | 75.8597 | 3.05E-18 | 7.29E-15 |
| *ENSG00000258757* | 10.96395 | 2.302171 | 73.00388 | 1.29E-17 | 2.82E-14 |
| *CPS1* | -8.10087 | 6.19761 | 70.90993 | 3.74E-17 | 7.46E-14 |
| *KDM5D* | 11.28027 | 2.112603 | 67.82162 | 1.79E-16 | 3.30E-13 |
| *QPRT* | 4.170247 | 5.894878 | 66.45008 | 3.59E-16 | 6.14E-13 |
| *PAX8-AS1* | 7.771922 | 5.274139 | 65.75099 | 5.12E-16 | 8.17E-13 |
| *MELTF* | 5.895153 | 5.681936 | 65.29405 | 6.45E-16 | 9.65E-13 |
| *AZGP1* | 4.485157 | 5.374802 | 63.9691 | 1.26E-15 | 1.78E-12 |
| *CX3CL1* | 4.60831 | 5.172242 | 63.58651 | 1.53E-15 | 2.04E-12 |
| *MST1L* | 4.83805 | 3.773323 | 63.3647 | 1.72E-15 | 2.16E-12 |
| *ANO1* | -4.67024 | 7.255916 | 59.58665 | 1.17E-14 | 1.40E-11 |
| *ABCC2* | 5.778894 | 3.409978 | 56.46563 | 5.72E-14 | 6.52E-11 |
| *HLA-DQB1* | 4.451044 | 7.423291 | 56.306 | 6.20E-14 | 6.75E-11 |

FC, fold change; CPM, counts per million; LR, likelihood ratio; FDR, false discovery rate.

*WDR72*, WD Repeat Domain 72; *XIST*, X Inactive Specific Transcript; *NOTUM*, Notum, Palmitoleoyl-Protein Carboxylesterase; *GNG4*, G Protein Subunit Gamma 4; *NKX6-3*, NK6 Homeobox 3; *GATA4*, GATA Binding Protein 4; *PAX6*, Paired Box 6; *KRT8P36*, Keratin 8 Pseudogene 36; *ENSG00000258757*, Novel Transcript, Antisense To ERO1L*; CPS1*, Carbamoyl-Phosphate Synthase 1; *KDM5D*, Lysine Demethylase 5D; *QPRT*, Quinolinate Phosphoribosyltransferase; *PAX8-AS1*, PAX8 Antisense RNA 1; *MELT*F, Melanotransferrin; *AZGP*1, Alpha-2-Glycoprotein 1, Zinc-Binding; *CX3CL1*, C-X3-C Motif Chemokine Ligand 1; *MST1L*, Macrophage Stimulating 1 Like (Pseudogene); *ANO1*, Anoctamin 1; *ABCC2*, ATP Binding Cassette Subfamily C Member 2; *HLA-DQB1*, Major Histocompatibility Complex, Class II, DQ Beta 1*.*
